# Weight Loss in Short-Term Interventions for Physical Activity and Nutrition Among Adults With Overweight or Obesity: A Systematic Review and Meta-Analysis

**DOI:** 10.5888/pcd21.230347

**Published:** 2024-04-04

**Authors:** Wendi Rotunda, Caroline Rains, Sara R. Jacobs, Valerie Ng, Rachael Lee, Stephanie Rutledge, Matt C. Jackson, Kristopher Myers

**Affiliations:** 1 RTI International, Research Triangle Park, North Carolina; 2 Centers for Disease Control and Prevention, Atlanta, Georgia; 3 CyberData Technologies, Herndon, Virginia

## Abstract

**Introduction:**

Reaching, enrolling, and retaining participants in lengthy lifestyle change interventions for weight loss is a major challenge. The objective of our meta-analysis was to investigate whether lifestyle interventions addressing nutrition and physical activity lasting 6 months or less are effective for weight loss.

**Methods:**

We searched for peer-reviewed studies on lifestyle change interventions of 6 months or less published from 2012 through 2023. Studies were screened based on inclusion criteria, including randomized controlled trials (RCTs) for adults with overweight or obesity. We used a random-effects model to pool the mean difference in weight loss between intervention and control groups. We also performed subgroup analyses by intervention length and control type.

**Results:**

Fourteen RCTs were identified and included in our review. Half had interventions lasting less than 13 weeks, and half lasted from 13 to 26 weeks. Seven were delivered remotely, 4 were delivered in person, and 3 used combined methods. The pooled mean difference in weight change was −2.59 kg (95% CI, −3.47 to −1.72). The pooled mean difference measured at the end of the intervention was −2.70 kg (95% CI, −3.69 to −1.71) among interventions lasting less than 13 weeks and −2.40 kg (95% CI, −4.44 to −0.37) among interventions of 13 to 26 weeks.

**Conclusion:**

Short-term multicomponent interventions involving physical activity and nutrition can achieve weight loss for adults with overweight or obesity. Offering short-term interventions as alternatives to long-term ones may reach people who otherwise would be unwilling or unable to enroll in or complete longer programs.

**Summary**

**What is already known on this topic?**

Long-term lifestyle change programs can be effective at achieving weight loss for adults with overweight or obesity and can lower their risks for developing chronic diseases, such as type 2 diabetes. However, enrollment and retention are challenging in long-term interventions.

**What is added by this report?**

We demonstrated that multicomponent nutrition and physical activity interventions of 6 months or less can achieve weight loss by the end of the intervention period.

**What are the implications for public health practice?**

Short-term lifestyle change programs can produce weight loss that may be associated with reduced risk of chronic diseases. Providing both short-term and long-term options could increase enrollment in such programs.

## Introduction

Approximately 60% of US adults have a chronic disease, and approximately 40% have 2 or more (1). Chronic diseases are a leading cause of death and disability (2) and contribute substantially to the $3.8 trillion in annual health care costs in the US (1). Multicomponent lifestyle change programs are known to be effective in reducing the risk of developing chronic diseases and largely focus on losing weight (3,4). Weight loss is an important objective for many lifestyle change interventions given the increased risk for people with overweight or obesity to develop chronic diseases, including type 2 diabetes (5), cardiovascular disease (6), and cancer (7). However, enrolling and remaining in such interventions are a challenge, particularly for those of longer duration (8,9). Thus, short-term interventions may have the potential to both enroll more participants and achieve higher retention (8,9). In addition, evidence indicates that most people achieve their greatest weight loss in the first 3 to 6 months of a lifestyle change intervention (10).

Previous systematic reviews examined interventions of various lengths for weight change (11–14) but did not look at whether the intervention length itself substantially affected body weight. Although 1 prior meta-analysis examined weight change in an intervention that lasted 6 months or less compared with 12 months or more, the study’s population was specific to adults with overweight or obesity who were also diagnosed with a mental illness (15). That analysis found, however, that the weight change effect size was similar in interventions of 6 months or less compared with interventions of 12 months or more.

Although weight loss is associated with preventing or delaying the onset of chronic conditions (3,4), long-term interventions have challenges in enrolling and retaining participants (8,9). We sought to understand the extent to which multicomponent interventions of 6 months or less were effective at achieving weight loss among adults with overweight or obesity.

## Methods

### Data sources

We searched PubMed via Medline, Web of Science, APA PsycInfo, Embase, CINAHL, and Cochrane Library for peer-reviewed studies on lifestyle change interventions of 6 months or less that were published from January 2012 through January 2023. We selected these years to ensure that the use of technology that might affect intervention length in the delivery of these interventions was reflected in the studies. Our search strategy (Table 1) used a combination of key terms including 1) a health condition or lifestyle and behavior term (eg, physical activity, overweight), 2) a program or intervention term (eg, lifestyle change, intervention), and 3) an outcome term (eg, weight loss). We also hand-searched systematic reviews identified in the searches.

**Table 1:** Search Strategy^a^: Systematic Review and Meta-Analysis of Weight Loss in Short-Term Physical Activity or Nutrition Interventions Among Adults With Overweight or Obesity, 2021–2023

Database	Strategy
PubMed	("hypertension"[Title] OR "blood pressure"[Title] OR "pre-hypertension"[Title] OR "uncontrolled blood pressure"[Title] OR "controlled blood pressure"[Title] OR "exercise"[Title] OR "physical activity"[Title] OR "aerobic fitness"[Title] OR "sedentary"[Title] OR "healthy diet"[Title] OR "nutrition"[Title] OR "healthy weight"[Title] OR "weight management"[Title] OR "diabetes"[Title] OR "manage"[Title] OR "prevent"[Title] OR "monitor"[Title] OR "health behavior"[Title] OR "self-monitoring"[Title] OR "blood pressure self-management"[Title] OR ("obesity"[Title] AND "Obesity"[Majr:NoExp]) OR "prediabetes"[Title] OR "muscle strengthening"[Title] OR "healthy eating"[Title] OR "pre-diabetes"[Title] OR ("Overweight"[Majr:NoExp] AND "overweight"[Title])) AND ("lifestyle change*"[Title] OR "life style change*"[Title] OR "National Diabetes Prevention Program*"[Title] OR "Diabetes Prevention Program*"[Title] OR "lifestyle modification*"[Title] OR "life style modification*"[Title] OR "lifestyle intervention*"[Title] OR "life style intervention*"[Title] OR "diabetes prevention"[Title] OR "diabetes risk reduction"[Title] OR "health promotion"[Title] OR "health program*"[Title] OR "health intervention*"[Title] OR "weight reduction program*"[Title] OR "weight loss intervention*"[Title] OR "weight loss program*"[Title] OR "weight control"[Title] OR "obesity management"[Title] OR "obesity prevention"[Title] OR "obesity control"[Title] OR "overweight management"[Title] OR "overweight prevention"[Title] OR "overweight control"[Title] OR "physical activity program*"[Title] OR "physical activity intervention*"[Title] OR "exercise program*"[Title] OR "exercise intervention*"[Title] OR "nutrition program*"[Title] OR "nutrition intervention*"[Title] OR intervention*[Title] OR "lifestyle program*"[Title] OR program*[Title] OR "YMCA"[Title] OR "behavioral lifestyle intervention*"[Title] OR "group session*"[Title] OR "goal setting"[Title] OR "behavioral counseling"[Title] OR "program champion*"[Title] OR "community health"[Title] OR "health coaching"[Title] OR "motivational interviewing"[Title] OR "group counseling"[Title] OR "primary prevention"[Title] OR "self-management"[Title] OR "disease management"[Title] OR ("weight management"[Title] AND "Obesity Management"[Majr:NoExp]) OR "health coach"[Title] OR "behavior modification"[Title]) AND ("weight loss outcome*"[Title/Abstract] OR "weight change"[Title/Abstract] OR "weight reduction outcome*"[Title/Abstract] OR "weight management outcome*"[Title/Abstract] OR (("BMI"[Title] OR "body mass index"[Title]) AND "Body Mass Index"[Majr]) OR ("weight loss"[Title] AND "Weight Loss"[Mesh:NoExp]) OR "weight reduction"[Title]) AND ("2012/01/01"[Date - Publication] : "3000"[Date - Publication]) Filters: English #2 #1 NOT ("Comment"[Publication Type] OR "Letter"[Publication Type] OR "Editorial"[Publication Type]) #3 #2 NOT (((teen*[Title] OR youth*[Title] OR adolescen*[Title] OR child*[Title]) NOT (adult[Title] OR adults[Title])) OR (("Adolescent"[Mesh] OR "Child"[Mesh] OR "Infant"[Mesh]) NOT "Adult"[Mesh]))

Web of Science	TI=("hypertension" OR "blood pressure" OR "pre-hypertension" OR "uncontrolled blood pressure" OR "controlled blood pressure" OR "exercise" OR "physical activity" OR "aerobic fitness" OR "sedentary" OR "healthy diet" OR "nutrition" OR "healthy weight" OR "weight management" OR "diabetes" OR "manage" OR "prevent" OR "monitor" OR "health behavior" OR "self-monitoring" OR "blood pressure self-management" OR "obesity" OR "prediabetes" OR "muscle strengthening" OR "healthy eating" OR "pre-diabetes" OR "overweight") AND TI=("lifestyle change*" OR "life style change*" OR "National Diabetes Prevention Program*" OR "Diabetes Prevention Program*" OR "lifestyle modification*" OR "life style modification*" OR "lifestyle intervention*" OR "life style intervention*" OR "diabetes prevention" OR "diabetes risk reduction" OR "health promotion" OR "health program*" OR "health intervention*" OR "weight reduction program*" OR "weight loss intervention*" OR "weight loss program*" OR "weight control" OR "obesity management" OR "obesity prevention" OR "obesity control" OR "overweight management" OR "overweight prevention" OR "overweight control" OR "physical activity program*" OR "physical activity intervention*" OR "exercise program*" OR "exercise intervention*" OR "nutrition program*" OR "nutrition intervention*" OR intervention* OR "lifestyle program*" OR program* OR "YMCA" OR "behavioral lifestyle intervention*" OR "group session*" OR "goal setting" OR "behavioral counseling" OR "program champion*" OR "community health" OR "health coaching" OR "motivational interviewing" OR "group counseling" OR "primary prevention" OR "self-management" OR "disease management" OR "weight management" OR "health coach" OR "behavior modification") AND (TI=("weight loss outcome*" OR "weight change" OR "weight reduction outcome*" OR "weight management outcome*" OR "weight reduction") OR (TI=("BMI" OR "body mass index") AND AK=("body mass index")) OR (TI=("weight loss") AND AK=("weight loss"))) and English (Languages) and Letters or Editorial Materials or Corrections (Exclude – Document Types) Timespan: 2012-01-01 to 2022-12-31 (Publication Date) #2 #1 NOT (TI=(teen* OR youth* OR adolescen* OR child*) NOT TI=(adult OR adults))

APA PsycInfo	TI ("hypertension" OR "blood pressure" OR "pre-hypertension" OR "uncontrolled blood pressure" OR "controlled blood pressure" OR "exercise" OR "physical activity" OR "aerobic fitness" OR "sedentary" OR "healthy diet" OR "nutrition" OR "healthy weight" OR "weight management" OR "diabetes" OR "manage" OR "prevent" OR "monitor" OR "health behavior" OR "self-monitoring" OR "blood pressure self-management" OR "obesity" OR "prediabetes" OR "muscle strengthening" OR "healthy eating" OR "pre-diabetes" OR "overweight") AND TI ("lifestyle change*" OR "life style change*" OR "National Diabetes Prevention Program*" OR "Diabetes Prevention Program*" OR "lifestyle modification*" OR "life style modification*" OR "lifestyle intervention*" OR "life style intervention*" OR "diabetes prevention" OR "diabetes risk reduction" OR "health promotion" OR "health program*" OR "health intervention*" OR "weight reduction program*" OR "weight loss intervention*" OR "weight loss program*" OR "weight control" OR "obesity management" OR "obesity prevention" OR "obesity control" OR "overweight management" OR "overweight prevention" OR "overweight control" OR "physical activity program*" OR "physical activity intervention*" OR "exercise program*" OR "exercise intervention*" OR "nutrition program*" OR "nutrition intervention*" OR intervention* OR "lifestyle program*" OR program* OR "YMCA" OR "behavioral lifestyle intervention*" OR "group session*" OR "goal setting" OR "behavioral counseling" OR "program champion*" OR "community health" OR "health coaching" OR "motivational interviewing" OR "group counseling" OR "primary prevention" OR "self-management" OR "disease management" OR "weight management" OR "health coach" OR "behavior modification") AND (TI ("weight loss outcome*" OR "weight change" OR "weight reduction outcome*" OR "weight management outcome*" OR "BMI" OR "body mass index" OR "weight reduction" OR "weight loss") OR AB ("weight loss outcome*" OR "weight change" OR "weight reduction outcome*" OR "weight management outcome*")) Limiters - Publication Year: 2012-2022; Peer Reviewed; English S2 S1 NOT (ZZ "comment/reply" OR ZZ "editorial" OR ZZ "letter") Limiters - Publication Year: 2012-2022; Peer Reviewed; English S3 S2 NOT (((TI teen* OR TI youth* OR TI adolescen* OR TI child*) NOT (TI adult OR TI adults)) OR ((ZG "adolescence (13-17 yrs)" OR ZG "childhood (birth-12 yrs)" OR ZG "infancy (2-23 mo)" OR ZG "neonatal (birth-1 mo)" OR ZG "preschool age (2-5 yrs)" OR ZG "school age (6-12 yrs)") NOT (ZG "adulthood (18 yrs & older)" OR ZG "aged (65 yrs & older)" OR ZG "middle age (40-64 yrs)" OR ZG "thirties (30-39 yrs)" OR ZG "very old (85 yrs & older)" OR ZG "young adulthood (18-29 yrs)"))) Limiters - Publication Year: 2012-2022; Peer Reviewed; English

Embase	("hypertension":ti OR "blood pressure":ti OR "pre-hypertension":ti OR "uncontrolled blood pressure":ti OR "controlled blood pressure":ti OR "exercise":ti OR "physical activity":ti OR "aerobic fitness":ti OR "sedentary":ti OR "healthy diet":ti OR "nutrition":ti OR "healthy weight":ti OR "weight management":ti OR "diabetes":ti OR "manage":ti OR "prevent":ti OR "monitor":ti OR "health behavior":ti OR "self-monitoring":ti OR "blood pressure self-management":ti OR ("obesity":ti AND 'obesity'/mj) OR "prediabetes":ti OR "muscle strengthening":ti OR "healthy eating":ti OR "pre-diabetes":ti OR ("overweight":ti AND 'obesity'/mj)) AND ("lifestyle change*":ti OR "life style change*":ti OR "National Diabetes Prevention Program*":ti OR "Diabetes Prevention Program*":ti OR "lifestyle modification*":ti OR "life style modification*":ti OR "lifestyle intervention*":ti OR "life style intervention*":ti OR "diabetes prevention":ti OR "diabetes risk reduction":ti OR "health promotion":ti OR "health program*":ti OR "health intervention*":ti OR "weight reduction program*":ti OR "weight loss intervention*":ti OR "weight loss program*":ti OR "weight control":ti OR "obesity management":ti OR "obesity prevention":ti OR "obesity control":ti OR "overweight management":ti OR "overweight prevention":ti OR "overweight control":ti OR "physical activity program*":ti OR "physical activity intervention*":ti OR "exercise program*":ti OR "exercise intervention*":ti OR "nutrition program*":ti OR "nutrition intervention*":ti OR intervention*:ti OR "lifestyle program*":ti OR program*:ti OR "YMCA":ti OR "behavioral lifestyle intervention*":ti OR "group session*":ti OR "goal setting":ti OR "behavioral counseling":ti OR "program champion*":ti OR "community health":ti OR "health coaching":ti OR "motivational interviewing":ti OR "group counseling":ti OR "primary prevention":ti OR "self-management":ti OR "disease management":ti OR ("weight management":ti AND 'obesity management'/mj) OR "health coach":ti OR "behavior modification":ti) AND ("weight loss outcome*":ti OR "weight change":ti OR "weight reduction outcome*":ti OR "weight management outcome*":ti OR (("BMI":ti OR "body mass index":ti) AND 'body mass'/mj) OR ("weight loss":ti AND 'body weight loss'/mj) OR "weight reduction":ti) AND [english]/lim AND [embase]/lim AND [2012-2022]/py #2 #1 NOT (Comment*:ti OR [editorial]/lim OR [letter]/lim) #3 #2 NOT (((teen*:ti OR youth*:ti OR adolescen*:ti OR child*:ti) NOT (adult:ti OR adults:ti)) OR (('adolescent'/exp OR 'child'/exp) NOT 'adult'/exp))

CINAHL	S1 (TI ("hypertension" OR "blood pressure" OR "pre-hypertension" OR "uncontrolled blood pressure" OR "controlled blood pressure" OR "exercise" OR "physical activity" OR "aerobic fitness" OR "sedentary" OR "healthy diet" OR "nutrition" OR "healthy weight" OR "weight management" OR "diabetes" OR "manage" OR "prevent" OR "monitor" OR "health behavior" OR "self-monitoring" OR "blood pressure self-management" OR "prediabetes" OR "muscle strengthening" OR "healthy eating" OR "pre-diabetes" OR "overweight") OR (MM "Obesity" AND TI "obesity")) AND (TI ("lifestyle change*" OR "life style change*" OR "National Diabetes Prevention Program*" OR "Diabetes Prevention Program*" OR "lifestyle modification*" OR "life style modification*" OR "lifestyle intervention*" OR "life style intervention*" OR "diabetes prevention" OR "diabetes risk reduction" OR "health promotion" OR "health program*" OR "health intervention*" OR "weight reduction program*" OR "weight loss intervention*" OR "weight loss program*" OR "weight control" OR "obesity management" OR "obesity prevention" OR "obesity control" OR "overweight management" OR "overweight prevention" OR "overweight control" OR "physical activity program*" OR "physical activity intervention*" OR "exercise program*" OR "exercise intervention*" OR "nutrition program*" OR "nutrition intervention*" OR intervention* OR "lifestyle program*" OR program* OR "YMCA" OR "behavioral lifestyle intervention*" OR "group session*" OR "goal setting" OR "behavioral counseling" OR "program champion*" OR "community health" OR "health coaching" OR "motivational interviewing" OR "group counseling" OR "primary prevention" OR "self-management" OR "disease management" OR "health coach" OR "behavior modification") OR (MM "Weight Control" AND TI "weight management")) AND (TI ("weight loss outcome*" OR "weight change" OR "weight reduction outcome*" OR "weight management outcome*") OR (MM "Body Mass Index" AND (TI "BMI" OR TI "body mass index")) OR (MM "Weight Loss" AND TI "weight loss") OR TI "weight reduction" OR AB ("weight loss outcome*" OR "weight change" OR "weight reduction outcome*" OR "weight management outcome*")) Limiters - Published Date: 20120101-20221231; English Language; Peer Reviewed; Exclude MEDLINE records S2 S1 NOT (ZT "commentary" OR ZT "editorial" OR ZT "letter" OR ZT "letter to the editor") Limiters - Published Date: 20120101-20221231; English Language; Peer Reviewed; Exclude MEDLINE records 188 S3 S2 NOT (((TI teen* OR TI youth* OR TI adolescen* OR TI child*) NOT (TI adult OR TI adults)) OR ((ZG "adolescent: 13-18 years" OR ZG "child, preschool: 2-5 years" OR ZG "child: 6-12 years" OR ZG "infant: 1-23 months") NOT (ZG "adult: 19-44 years" OR ZG "aged, 80 & over" OR ZG "aged: 65+ years" OR ZG "middle aged: 45-64 years"))) Limiters - Published Date: 20120101-20221231; English Language; Peer Reviewed; Exclude MEDLINE records

Cochrane Library	#1 ("hypertension" OR "blood pressure" OR "pre-hypertension" OR "uncontrolled blood pressure" OR "controlled blood pressure" OR "exercise" OR "physical activity" OR "aerobic fitness" OR "sedentary" OR "healthy diet" OR "nutrition" OR "healthy weight" OR "weight management" OR "diabetes" OR "manage" OR "prevent" OR "monitor" OR "health behavior" OR "self-monitoring" OR "blood pressure self-management" OR "prediabetes" OR "muscle strengthening" OR "healthy eating" OR "pre-diabetes"):ti OR ([mh obesity[mj]] AND ("obesity"):ti) OR ([mh overweight[mj]] AND ("overweight"):ti) #2 (lifestyle NEXT change* OR "life style" NEXT change* OR "National Diabetes Prevention" NEXT Program* OR "Diabetes Prevention" NEXT Program* OR lifestyle NEXT modification* OR "life style" NEXT modification* OR lifestyle NEXT intervention* OR "life style" NEXT intervention* OR "diabetes prevention" OR "diabetes risk reduction" OR "health promotion" OR health NEXT program* OR health NEXT intervention* OR "weight reduction" NEXT program* OR "weight loss" NEXT intervention* OR "weight loss" NEXT program* OR "weight control" OR "obesity management" OR "obesity prevention" OR "obesity control" OR "overweight management" OR "overweight prevention" OR "overweight control" OR "physical activity" NEXT program* OR "physical activity" NEXT intervention* OR exercise NEXT program* OR exercise NEXT intervention* OR nutrition NEXT program* OR nutrition NEXT intervention* OR intervention* OR lifestyle NEXT program* OR program* OR "YMCA" OR "behavioral lifestyle" NEXT intervention* OR group NEXT session* OR "goal setting" OR "behavioral counseling" OR program NEXT champion* OR "community health" OR "health coaching" OR "motivational interviewing" OR "group counseling" OR "primary prevention" OR "self-management" OR "disease management" OR "health coach" OR "behavior modification"):ti OR ([mh "obesity management"[mj]] AND ("weight management"):ti) #3 ("weight loss" NEXT outcome* OR "weight change" OR "weight reduction" NEXT outcome* OR "weight management" NEXT outcome* OR "weight reduction" OR "BMI" OR "body mass index"):ti OR (("weight loss"):ti AND [mh "Weight Loss"]) #4 (comment* OR letter OR editorial): #5 ((teen* OR youth* OR adolescen* OR child*):ti NOT (adult OR adults):ti) OR (([mh Adolescent] OR [mh Child] OR [mh Infant]) NOT [mh Adult]) #6 (#1 AND #2 AND #3) NOT (#4 OR #5) with Cochrane Library publication date from Jan 2012 to Dec 2022 with Publication Year from 2012 to 2022

a This search strategy was initially developed as part of a broader systematic review.

### Study selection

We included peer-reviewed primary research studies published in English that reported on lifestyle change interventions of 6 months or less (operationalized as 26 weeks) for adults aged 18 years or older with overweight or obesity. Studies had to report weight loss outcomes to be eligible for inclusion. We excluded studies in which participants were already diagnosed with a chronic condition, such as hypertension or diabetes, but included studies that were intended for populations with heightened risks for developing chronic conditions. We also excluded studies without an intervention component focused on nutrition or physical activity.

We included randomized controlled trials (RCTs) only and excluded other study designs, such as observational studies, given that other designs are more susceptible to bias or confounding, and studies that did not conduct an intention-to-treat analysis, because complete case analysis may lead to bias in the intervention effect estimates (16). Studies also had to be conducted in countries rated as very high in development based on the United Nations Human Development Index (17), so that findings would be more generalizable to US adults with overweight or obesity.

For studies with multiple intervention arms, we selected a primary arm to include in the analysis. We selected the primary intervention arm based on several factors, such as the intervention included either nutrition or physical activity with the goal of weight loss (some of the alternative interventions did not include a lifestyle change component) or the intervention included multiple methods such as in-person sessions and an online forum meant to maximize participation and retention. In cases where multiple intervention arms met the above criteria, we included 1 intervention arm in the main analysis and the other intervention arm in a sensitivity analysis.

### Data extraction and critical appraisal

We used Covidence Systematic Review Software (Veritas Health Innovation) to help manage the systematic review process. Two team members used the study selection criteria to independently review each title and abstract. All conflicts at the title and abstract stage were advanced to the full-text review. Full-text articles were also reviewed independently by 2 reviewers. Conflicts were resolved by a third senior reviewer, who also confirmed inclusion of all final articles.

Reviewers used a standardized extraction form to extract key data. The extraction form was programmed in REDCap (REDCap Consortium) (18,19), and each article was extracted by one reviewer and checked for accuracy by a senior reviewer. Data on body weight change were extracted in the reported units, either kilograms or pounds, and then standardized into kilograms for all studies. We used the National Heart, Lung, and Blood Institute’s Study Quality Assessment Tool (Box) (20) to document the methodologic quality of the included studies. Studies were scored and classified as poor (0–5 points), fair (6–12 points), or high (13,14 points). All 5 reviewers were trained on the extraction and study quality assessment tools before they completed the full-text extractions.

**Box. National Heart Lung, and Blood Institute’s Study Quality Assessment Tool (https://www.nhlbi.nih.gov/health-topics/study-quality-assessment-tools)**

**Answer options are yes, no, neither (cannot determine, not reported, or not applicable)**

1. Was the study described as randomized, a randomized trial, a randomized clinical trial, or an RCT?

2. Was the method of randomization adequate (ie, use of randomly generated assignment)?

3. Was the treatment allocation concealed (so that assignments could not be predicted)?

4. Were study participants and providers blinded to treatment group assignment?

5. Were the people assessing the outcomes blinded to the participants' group assignments?

6. Were the groups similar at baseline on important characteristics that could affect outcomes (eg, demographics, risk factors, comorbid conditions)?

7. Was the overall dropout rate from the study at endpoint 20% or lower of the number allocated to treatment?

8. Was the differential dropout rate (between treatment groups) at endpoint 15 percentage points or lower?

9. Was there high adherence to the intervention protocols for each treatment group?

10. Were other interventions avoided or similar in the groups (eg, similar background treatments)?

11. Were outcomes assessed using valid and reliable measures, implemented consistently across all study participants?

12. Did the authors report that the sample size was sufficiently large to be able to detect a difference in the main outcome between groups with at least 80% power?

13. Were outcomes reported or subgroups analyzed prespecified (ie, identified before analyses were conducted)?

14. Were all randomized participants analyzed in the group to which they were originally assigned (ie, did they use an intention-to-treat analysis)?

### Statistical analysis

We used the mean body weight change from baseline to the end of the intervention time point for both the intervention and comparison groups. When these data were not reported, we used other data provided in the study for calculating the change (21). We used Stata, version 17 (StataCorp LLC) to calculate the pooled mean difference in weight change (in kilograms) by using a random effects model with the inverse variance weighting method described by DerSimonian and Laird (22).

We assessed statistical heterogeneity (ie, variability resulting from differences in the study effects) in pooled estimates by examining *I* ^2^ statistics and *P* values. We considered *I* ^2^ values of 0% to 40% to indicate unimportant heterogeneity, 30% to 60% to indicate moderate heterogeneity, 50% to 90% to indicate substantial heterogeneity, and 75% to 100% to indicate considerable heterogeneity (23). When we observed moderate, substantial, or considerable heterogeneity (23), we conducted sensitivity analyses after removing outlier studies. We also visually examined plots for effects of different study characteristics and intervention factors, including the intervention method, proportion of female participants, average age of participants, average baseline weight of participants, and the percentage of participants completing the intervention.

Subgroup analyses were performed based on intervention length (<13 wk or 13–26 wk) and the type of comparison group described as low touch, usual care, or wait list. Low-touch comparison groups could entail a minimal amount of intervention for lifestyle change; for example, participants may have received informative emails (24) or printed information related to healthy habit formation (25). Usual-care groups were encouraged to engage in their regular behaviors without changing their usual routine. Wait-list or clinical-care comparison groups would eventually receive the intervention after data collection. We made the distinction between groups because a comparison group that included some engagement with participants could limit the ability to detect true intervention effects on weight loss compared with comparison groups that were considered usual care or were delayed in receiving the intervention.

## Results

### Study characteristics

**Figure 1:**
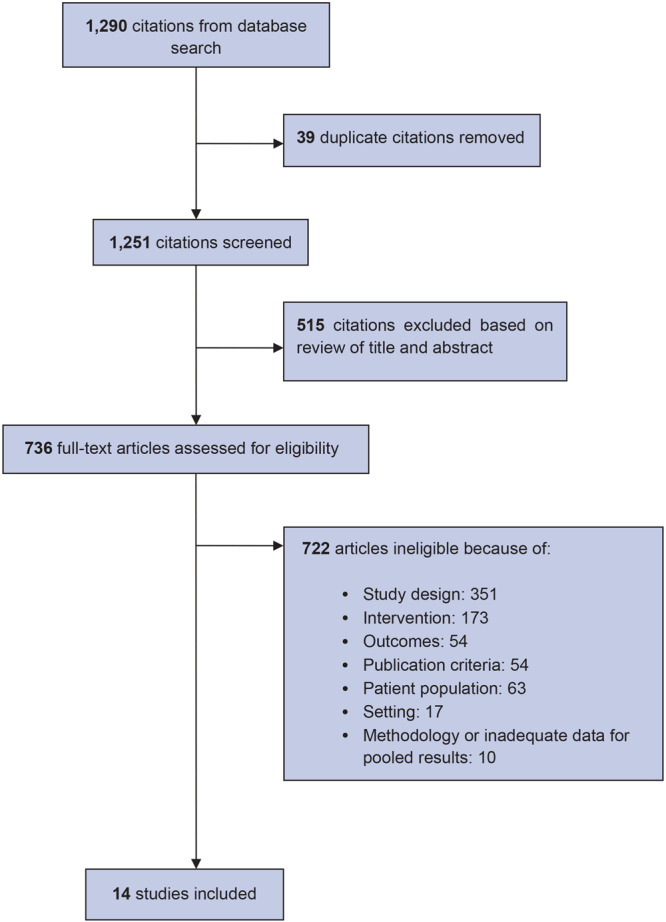
Flowchart of steps in selection of 14 studies for inclusion in a systematic review of weight loss in short-term interventions for physical activity and nutrition among adults with overweight or obesity.

We screened 1,251 unique citations and identified 14 RCTs for inclusion in our review (Figure 1). Among the 14 studies included, half had a wait-list comparison group (26–32), 5 had low-touch comparison groups (24,25,33–35), and 2 had usual-care comparison groups (36,37).

Five of the 14 studies were conducted in the US (26,28,32,34,35), 5 in Australia (27,29–31,36), 2 in the United Kingdom (33,37), 1 in Canada (25), and 1 in Turkey (24) (Table 2). Seven studies had interventions lasting less than 13 weeks with a median of 12 weeks (24,25,27–31), and the other 7 studies lasted from 13 to 26 weeks, with a median of 24 weeks (26,32–37). The average age of study participants ranged from 40 to 52 years (24,25,27–31). Two studies included only women (28,35), and 3 studies included only men (29,30,32). Average baseline weight of study participants across all studies ranged from 82 kg to 139 kg. Seven of the 14 studies were delivered virtually, which included the use of websites, telephone, and email (24,29–31,34,36,37); 3 were a mix of both virtual and in-person components (27,32,35); and 4 were delivered exclusively in person (25,26,28,33). Among the 7 in-person and mixed-delivery intervention arms, 2 were conducted in a health care setting (28,33), 2 were conducted in a community setting (25,26), 1 was conducted in a university setting (27), 1 was conducted in the workplace (35), and 1 did not specify a setting (32).

**Table 2:** Characteristics of Study Interventions and Participants, Systematic Review and Meta-Analysis of Weight Loss in Short-Term Physical Activity or Nutrition Interventions Among Adults With Overweight or Obesity, 2021–2023

Author, year, country	Study type and setting	Population, inclusion criteria	Sample size, intervention and control groups at trial start and end; weight change, kg	Intervention group description and focus	Control group description	Intervention duration and intensity	Study quality rating
Arterburn et al, 2019, US (34)	Online or distance learning via telephone	Participants aged 18–65 y, BMI (kg/m^2^) 27–42, interested in losing weight and at stable weight	Start: intervention, n = 66, control, n = 67. End: intervention, n = 63, control n = 61. Weight change: intervention, n = −5.00; control, n = −0.20	Focus: Nutrition. Intervention group was instructed to follow a specific program, intended to provide 800–1,000 kcal/d. Support tools: Written program materials (meal plan guide, food journals, recipes, habits of health book, and workbook). Also had access to online support tools and the intervention’s nutrition support team. Suggested exercise: Participants were encouraged, but not required, to exercise. Group or individual class: Participants were assigned a coach to have one-on-one coaching via telephone.	Control group followed a self-directed, reduced calorie diet. Participants were instructed to log food, exercise, and progress; received a one-on-one introduction session; and received other publicly available information from ChooseMyPlate.com.	Duration: 16 weeks. Intensity: 23 coaching telephone calls	Fair
Baetge et al, 2017, US (28)	In-person: health care setting	Women aged 18–69 y, sedentary, with overweight or obesity, no diabetes diagnosis, and not in a diet or exercise program within 3 months of starting the study	Start: intervention, n = 29, control, n = 20. End: intervention, n = 29, control, n = 20. Weight change: intervention, n = −4.26; control, n = −0.10	Focus: Both nutrition and physical activity. Intervention group participants had an initial 1,200 kcal/d goal for the first week to 1,500 kcal/d thereafter and supervised circuit training. Educational resources: Participants were given a commercial weight management plan book. Organized activity: Required in-person exercise training sessions. Support tools: Participants were given food and exercise diaries, meal replacements, and supplements.	Control group was wait-listed and instructed not to change their dietary intake or engage in physical activity for the 12 weeks of the study, but were randomized into 1 of the 4 diet groups once they completed their initial control group.	12 weeks. Intensity: 4 supervised circuit-training sessions per week	Fair
Carnie et al, 2013, US (35)	Mixed: In-person, workplace; online, web-based application	Women employees of National Institutes of Health with BMI ≥25, no diabetes diagnosis, healthy, with no active participation in other exercise or weight loss programs	Start: intervention, n = 99; control, n = 100. End: intervention, n = 69; control, n = 70. Weight change: intervention, n = −2.7; control. n = −2.0	Focus: Nutrition. Personalized goals: A dietitian provided participants a daily meal plan reflecting individualized calorie needs. Group and individual class: One-on-one coaching with dietitians and group nutrition education sessions. Educational resources: Access to a website that included resources to promote exercise and healthy eating. Support tools: Access to private fitness rooms, a pedometer.	Control group was provided wellness information on the internet.	24 weeks. Weekly group nutrition education sessions for the first 3 months, monthly for the last 3 months	Low^a^
Cleo et al, 2019, Australia (27)	Mixed: In-person: university; Online: email	Participants aged 18–75 y, able to consent, BMI ≥25, could attend all required appointments, had access to email or telephone, and free from exercise-limiting comorbidities	Start: intervention, n = 25; control, n = 25. End: intervention, n = 21; control, n = 23. Weight change: intervention, n = −3.30; control, n = −0.40	Focus: Both nutrition and physical activity. Educational resources: Received a leaflet that listed 7 behaviors associated with negative energy balance, 2 behaviors designed to improve awareness of food intake, and 1 behavior to promote routine establishment. Support tools: A logbook was provided to participants for recording adherence to the 7 behaviors. Group/individual classes: group introduction meeting.	Control group was wait-listed. They were contacted weekly via telephone for 12 weeks and received no weight-loss advice. They were later offered either of the interventions after this time.	12 weeks. Single 2-h group introduction meeting	Fair
Collins et al, 2012, Australia (30)	Online or distance learning via a web-based application	Adults aged 18–60 y, BMI 25–40, not participating in other weight loss programs, passed a health screen, available for in-person assessments, and had internet, computer, and email access	Start: intervention, n = 106; control, n = 104. End: intervention, n = 90; control, n = 96. Weight change: intervention, n = −2.98; control, n = 0.36	Focus: Both nutrition and physical activity. Personalized goals: Received personalized daily calorie targets and menu plans. Personalized messages: Received personalized messages based on whether participant had accessed site or recorded weight. Received personalized feedback: Personalized feedback from physical activity and nutrition diaries and summaries and visualization of weight loss achievements. Automatic messages: Received automated e-feedback. Educational resources: Access to program website. Support tools: Received a reminder schedule to use the diary. Peer mentoring: Participation in web-based community forums.	Control group was wait-listed.	12 weeks, new content weekly	High
Crane et al, 2015, US (32)	Mixed. In-person: not specified; Online: web-based applications	Men aged 18–65 y, BMI 25–40, with regular internet access, and able to exercise safely	Start: intervention, n = 53; control, n = 54. End: intervention, n = 48; control, n = 49. Weight change: intervention, n = −5.10; control, n = −0.50	Focus: Both nutrition and physical activity. Intervention group participated in a program and encouraged to implement modest calorie restriction via 100-calorie reductions and established physical activity progression. Personalized messages: Automated, tailored feedback was provided based on meeting or not meeting physical activity goal, days of daily weighing, weight loss, and changes made to eating. Personalized goals: Encouraged participants to personalize the exercise types with gradual progression and plans and mastering one dietary change at a time. Educational resources: Participants self-tailored the order of specific diet strategies to focus on each week and received a brief online lesson on how to implement the strategy. Automatic messages: A reminder to complete the contact was sent mid-week. Individual/group sessions: Two face-to-face sessions.	Control group was wait-listed.	22 weeks. Two 1-h face-to-face group sessions followed by interactive online intervention contacts weekly for 10 wks and monthly online contact for 3 mo. Encouraged gradual physical activity progression to 225 min/wk.	Fair
Hardcastle et al, 2013, UK (33)	In-person: health care setting	Participants aged 18-65 y, BMI ≥28, and ≥1 CVD risk factors	Start: intervention, n = 203; control, n = 131. End: intervention, n = 125; control, n = 93. Weight change: intervention, n = −0.62; control, n = 0.13	Focus: Both nutrition and physical activity. Intervention group received face-to-face consultation with a physical activity specialist or registered dietician, and goal setting. Personal goals: Recommendation to be physically active for 30 minutes, at least 5 times a week. Educational resources: Participants received exercise and nutrition information. Group/individual sessions: Five face-to-face motivational interviewing sessions delivered by a physical activity specialist and registered dietician.	Control group received a leaflet with lifestyle guidelines, the physiological and psychological benefits of increased physical activity, and a food and physical activity quiz with advice depending on scores.	26 wk. Up to 5 face-to-face motivational interviewing sessions conducted over 6 mo; each session was approximately 20–30 min. Recommendation for 150 min physical activity per week.	Low^a^
Hepdurgun et al, 2020, Turkey (24)	Online or distance learning: web-based application	Participants aged 18–65 y, BMI 25–40, and internet access	Start: intervention, n = 51; control, n = 50. End: intervention, n = 40; control, n = 36. Weight change: intervention, n = −2.28; control, n = −0.74	Focus: Both nutrition and physical activity. Intervention group received access to the internet-based program, weekly lesson videos, and food diaries; were given feedback on their performance and sent reminder emails if inactive. Personalized messages: Participants were given feedback on their performance of watching videos and filling their food diary through the in-program message system. Educational resources: 69 videos prepared related to nutrition with information on various topics such as healthy eating, and 145 videos of exercises that can be done at home. Support tools: Food diary to follow up on the daily calorie intake and change in body weight. Automatic messages: Reminder messages were sent if participants had not logged in for a week.	Control group received an initial visit, weekly informative emails about healthy eating, physical exercise, and weight loss.	8 weeks	Low^a^
Jane et al, 2018, Australia (36)	Online or distance learning: web-based application	Participants aged 21-65 y with BMI 25-40	Start: intervention, n = 46; control, n = 45. End: intervention, n = 19; control, n = 17. Weight change: intervention, n = −4.60; control, n = 0.20	Focus: Both nutrition and physical activity. Intervention group received instructions to follow a specific diet and peer support through a Facebook group. Educational resources: Participants received example breakfast, lunch, and dinner recipes. Suggest exercise: Recommends individuals aim for a step count of 10,000 steps per day. Peer mentoring: peer support from other group members (the Facebook group).	Control group was instructed to follow the Australian government’s dietary guidelines and the National Physical Activity Guidelines for Adults as standard care.	24 weeks. Clinical appointments for a duration of approximately 15 min at baseline, and weeks 6, 12, 18 and 24. Suggested step count of 10,000 steps per day.	Low^a^
Mayer et al, 2019, US (26)	In-person: community-based setting	Residents of East Harlem who speak Spanish- or English, BMI ≥25, and no diabetes diagnosis	Start: intervention, n = 210; control, n = 192. End: intervention, n = 156, control, n = 147. Weight change: intervention, n = −1.20; control, n = −0.50	Focus: Both nutrition and physical activity. Support tools: Participants made “action plans” and provided peer support by exercising together and brainstorming ideas to address challenges they identified. Peer mentoring: Peer-led workshops at community sites. Individual/group sessions: Group peer-led workshops.	Control group was wait-listed. They received written materials in English and Spanish about diabetes prevention and a copy of their test results, and were invited to take part in the peer education workshops at no cost after a 1-year waiting period.	24 weeks; eight 90-min peer-led workshops	Low^a^
Morgan et al, 2012, Australia (31)	Online or distance learning in web-based application	Male employees of Tomago Aluminium aged 18-65 y, BMI 25-40	Start: intervention, n = 65; control, n = 45. End: (data missing) Weight change: intervention, n = −4.00; control, n = −0.30	Focus: Both nutrition and physical activity. Intervention group received a face-to-face information session, access to a study website to report weight and daily eating and exercise diaries, weight-loss education resources, a pedometer, and financial incentives. Educational resources: Overview of basic education for weight loss. Support tools: A study website, resources consisting of a weight loss handbook, a website user guide, and a pedometer. Incentives: A $50 gift voucher per crew member to be spent at a local sporting equipment store for the crew with the highest mean percentage weight loss after 1 month and at the conclusion of the program. Group/individual class: Face-to-face intervention session.	Control group was wait-listed for 14 weeks.	12 weeks. One face-to-face information session (75 min)	High
Morgan et al, 2013, Australia (29)	Online or distance learning in web-based application	Men with BMI 25-40, from the Hunter Region of New South Wales, Australia, with access to a mobile phone and a computer with email and internet	Start: intervention, n = 53 control, n = 52. End: intervention, n = 43 control, n = 45 Weight change: intervention, −4.40 control, n = −0.50	Focus: Both nutrition and physical activity. Intervention group received a website user guide, an online food and exercise diary, and feedback emails about their food and exercise diary entries. Personalized messages: Received 7 feedback emails about their food and exercise diary entries. Personalized goals: Advised to achieve a negative energy balance of 2000 kilojoules (kJ) per day. Men were asked to set 3 SMART goals each month (one each for weight, physical activity, and eating). Support tools: Pedometer, tape measure for waist circumference, a kilojoule counter book, online food and exercise diary. Educational resources: A weight loss DVD, a weight loss handbook and support guide, and a website user guide.	Control group was wait-listed. They received no program until after the 6-month assessments.	12 weeks. Participants to weigh-in -- record their weight on the website at least once a week	High
Padwal et al, 2017, Canada (25)	In-person: community-based setting	Adults with BMI ≥35	Start: intervention, n = 215; control, n = 211. End: intervention, n = 215; control, n = 211. Weight change: intervention, n = −3.70; control, n = −2.90	Focus: Both nutrition and physical activity. Intervention groups received group education sessions for healthful eating, increasing physical activity, goal setting, and self-monitoring. Group/individual sessions: 13 group sessions.	Control group received printed educational materials. After a week, they were contacted to be encouraged to read the printed materials, register for in-person sessions, or access the web modules.	12 weeks. 13 group sessions	Low^a^
Yardley et al, 2014, UK (37)	Online or distance learning in web-based application and telephone	Adults aged ≥18 y and BMI ≥30	Start: intervention, n = 47; control, n = 43. End: intervention, n = 37 control, n = 29. Weight change: intervention, n = −4.23; control, n = −1.99	Focus: Both nutrition and physical activity. The web-based intervention was designed to provide support for self-management of weight, based on patient choice of either a low-calorie or a low- carbohydrate eating plan and encouragement of physical activity. Personalized messages: Users received advice based on progress (eg, positive feedback if successful, advice on overcoming barriers if unsuccessful). Educational resources: Recommended links to other relevant high-quality websites. Support tools: A web-based goal-setting tool required patients to choose their first weekly goal from preset choices likely to promote significant weight loss; could set motivational automated messages and track weight and calories. Group/individual sessions: one-on-one contact in face-to-face with nurse.	Control group received access to interventions and support as determined by the primary care staff and was offered access to the website at the end of the study.	26 weeks. Scheduled support at 2 weeks and then monthly for the 6 months of the study (a total of 7 contacts)	Low^a^

Abbreviations: I, intervention; C, comparator; BMI, body mass index; CVD, cardiovascular disease; NIH, National Institutes of Health; UK, United Kingdom.

a Study was determined to be low quality because of high participant attrition (>20%).

For most of the 14 studies, the intervention focus was on both improving nutrition and increasing physical activity. Two interventions focused only on improving nutrition (34,35). Across the 14 studies that included a focus on nutrition, 7 described the nutritional component (28–30,32,34–36), 3 recommended a specific caloric value (eg, 1,200 calories per day) (28,29,34), 3 recommended participant-tailored guidance regarding calorie intake (30,32,35), and 1 allocated participants to a specific diet (36). Four studies described the physical activity component (28,32,33,36). One study reported structured group exercise in the form of 4 supervised circuit training sessions per week (28), and 3 reported providing participants with physical activity minute-count or step-count goals (32,33,36).

All 14 interventions also included more than 1 intervention component. For example, participants in 1 study (31) received a face-to-face information session, access to a study website to report daily diet and exercise, weight-loss education resources, a pedometer, and financial incentives. Another study (24) provided components that included access to an internet-based program, weekly lesson videos, food diaries, and both personalized and automatic messages. Eleven of the 14 studies provided participants with educational resources, such as booklets or access to information on a website (24,27–33,35–37); 10 studies provided participants with support tools, such as pedometers, scales, or access to food tracking logs (24,26–31,34,35,37); and 9 studies offered group or individual classes (25–27,31–35,37). Five studies supported participants by helping them set personalized exercise or calorie intake goals (29,30,32,33,35), 3 provided peer mentors or access to online discussion boards or forums where participants could work with other participants (26,30,36), and 3 sent automatic messages to participants that were not personalized, for example, messages reminding them to exercise or keep up their goals (24,30,32). In addition, 1 study provided financial incentives to participants who were part of a cohort that achieved the highest mean percentage weight loss after 1 month and at the end of the intervention (31).

### Weight change

**Figure 2:**
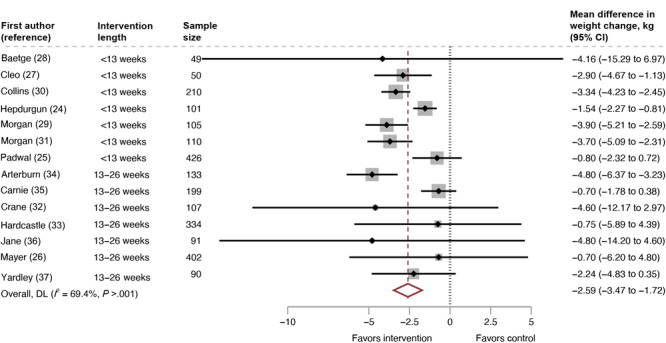
Mean difference in weight change across all included studies, intervention versus control, systematic review of weight loss in short-term interventions (N = 14) for physical activity and nutrition among adults with overweight or obesity. Meta-analysis was of the effects of intervention versus control on mean difference in weight change (kg) among the 14 included studies. Values less than 0 indicate an intervention effect (ie, favors intervention), and values greater than 0 indicate no intervention effect (ie, favors control). Abbreviation: DL, DerSimonian and Laird’s Q test (22).

**Figure 3:**
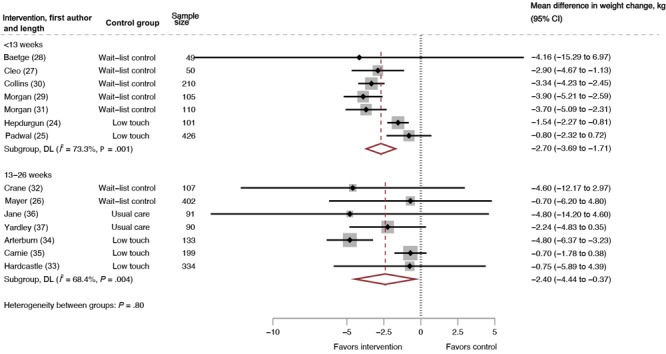
Mean difference in weight change by intervention duration, intervention versus control, systematic review of weight loss in short-term interventions (N = 14) for physical activity and nutrition among adults with overweight or obesity. Meta-analysis was of the effects of the intervention versus control on mean difference in weight change (kg), stratified by intervention duration. Intervention duration is defined as less than 13 weeks or 13 to 26 weeks. Values less than 0 indicate an intervention effect (ie, favors intervention), and values greater than 0 indicate no intervention effect (ie, favors control). Abbreviation: DL, DerSimonian and Laird’s Q test (22). Overall, DL (*I* ^2^ = 69.4%,* P* >.001).

The pooled mean difference for weight change was less than −2.59 kg (95% CI, −3.47 to −1.72; 14 RCTs; 2,407 participants; *I* ^2^ = 69%) (Figure 2). The negative difference in mean weight change indicates that people in the intervention groups lost more weight than those in the comparison groups. For the studies with interventions lasting less than 13 weeks, the pooled mean difference for weight change was −2.70 kg (95% CI, −3.69 to −1.71; 7 RCTs, 1,051 participants, *I* ^2^ = 73%). For the studies with interventions lasting 13 to 26 weeks, the pooled mean difference for weight change was −2.40 kg (95% CI, −4.44 to −0.37; 7 RCTs, 1,356 participants, *I* ^2^ = 69%) (Figure 3). We conducted a moderator analysis with intervention duration and found a significant difference based on intervention duration (*P* =.046).

**Table:** 

First author (reference)	Intervention length, weeks	Sample size	Mean difference in weight change, kg (95% CI)
Baetge (28)	<13	49	–4.16 (–15.29 to 6.97)
Cleo(27)	<13	50	–2.90 (–4.67 to –1.13)
Collins (30)	<13	210	–3.34 (–4.23 to –2.45)
Hepdurgun (24)	<13	101	–1.54 (–2.27 to –0.81)
Morgan (29)	<13	105	–3.90 (–5.21 to –2.59)
Morgan (31)	<13	110	–3.70 (–5.09 to –2.31)
Padwal (25)	13–26	426	–0.80 (–2.32 to 0.72)
Arterburn (34)	13–26	133	–4.80 (–6.37 to –3.23)
Carnie (35)	13–26	199	–0.70 (–1.78 to 0.38)
Crane (32)	13–26	107	–4.60 (–12.17 to 2.97)
Hardcastle (33)	13–26	334	–0.75 (–5.89 to 4.39)
Jane (36)	13–26	91	–4.80 (–14.20 to 4.60)
Mayer (26)	13–26	402	–0.70 (–6.20 to 4.80)
Yardley (37)	13–26	90	–2.24 (–4.83 to 0.35)

**Table:** 

First author (reference)	Intervention length, weeks	Control	Sample size	Mean difference in weight change, kg (95% CI)
Baetge (28)	<13	Wait-list control	49	–4.16 (–15.29 to 6.97)
Cleo (27)	<13	Wait-list control	50	–2.90 (–4.67 to –1.13)
Collins (30)	<13	Wait-list control	210	–3.34 (–4.23 to –2.45)
Morgan (29)	<13	Wait-list control	105	–3.90 (–5.21 to –2.59)
Morgan (31)	<13	Wait-list control	110	–3.70 (–5.09 to –2.31)
Hepdurgun (24)	<13	Low touch	101	–1.54 (–2.27 to –0.81)
Padwal (25)	<13	Low touch	426	–0.80 (–2.32 to 0.72)
Crane (32)	13–26	Wait-list control	107	–4.60 (–12.17 to 2.97)
Mayer (26)	13–26	Wait-list control	402	–0.70 (–6.20 to 4.80)
Jane (36)	13–26	Usual care	91	–4.80 (–14.20 to 4.60)
Yardley (37)	13–26	Usual care	90	–2.24 (–4.83 to 0.35)
Arterburn (34)	13–26	Low touch	133	–4.80 (–6.37 to –3.23)
Carnie (35)	13–26	Low touch	199	–0.70 (–1.78 to 0.38)
Hardcastle (33)	13–26	Low touch	334	–0.75 (–5.89 to 4.39)

### Heterogeneity and sensitivity analyses

**Figure 4:**
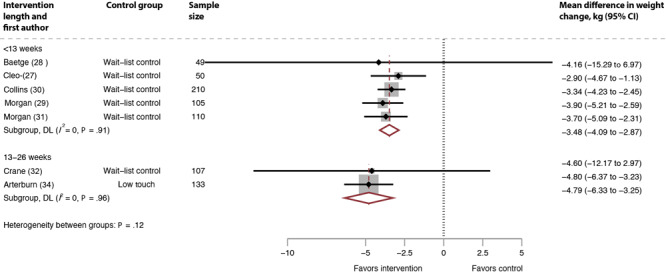
Mean difference in weight change, systematic review of weight loss in short-term interventions (N = 14) for physical activity and nutrition among adults with overweight or obesity, sensitivity analysis removing studies with high attrition, intervention versus control. Meta-analysis was of the effects of the intervention versus control on mean difference in weight change (kg), removing the studies with high attrition (24–26,30,33,35–37) as a sensitivity analysis. Values less than 0 indicate an intervention effect (ie, favors intervention), and values greater than 0 indicate no intervention effect (ie, favors control).

The pooled results had substantial heterogeneity overall and when stratified by intervention duration. We conducted a sensitivity analysis by removing studies with high attrition (> 20%) (24–26,33,35–37) (Figure 4). From the 7 studies with interventions of less than 13 weeks, we dropped 2 low-touch comparison group studies with high attrition (24,25). Among the 5 remaining studies, heterogeneity improved (*I* ^2^ = 0%, *P* =.91) and resulted in a larger mean difference for weight change: −3.48 kg (95% CI, −4.09 to −2.87). From the 7 studies with interventions of 13 to 26 weeks, we dropped 1 wait-list control study (26), 2 low-touch comparison group studies (33,35), and 2 usual-care comparison group studies (36,37) with high attrition. Among the 2 remaining studies (32,34), heterogeneity improved (*I* ^2^ = 0%, *P* = .97) and resulted in a larger mean difference for weight change: −4.79 kg (95% CI: −6.30 to −3.25).

**Table:** 

First author (reference)	Intervention length, weeks	Control group	Sample size	Mean difference in weight change, kg (95% CI)
Baetge (28)	<13	Wait-list control	49	–4.16 (–15.29 to 6.97)
Cleo (27)	<13	Wait-list control	50	–2.90 (–4.67 to –1.13)
Collins (30)	<13	Wait-list control	210	–3.34 (–4.23 to –2.45)
Morgan (29)	<13	Wait-list control	105	–3.90 (–5.21 to –2.59)
Morgan (31)	<13	Wait-list control	110	–3.70 (–5.09 to –2.31)
Crane (32)	13–26	Wait-list control	107	–4.60 (–12.17 to 2.97)
Arterburn (34)	13–26	Low touch	133	–4.80 (–6.37 to –3.23)

We also assessed heterogeneity by intervention method and participant characteristics. We examined results by delivery method (in person, online or other distance learning, or mixed) and gender of study participants (all men, >75% women, or a balanced mix of men and women). For results by delivery method, we found that the 7 studies conducted via online or other distance learning (24,29–31,34,36,37) had a larger effect size (−3.26 kg, *I* ^2^ = 75%) compared with the 3 studies conducted by using both online or other distance learning and in-person components (27,32,35) (−1.85 kg, *I* ^2^ = 60%), and the 4 studies conducted in person (25,26,28,33) (−0.84 kg, *I* ^2^ = 0%). We also found that for results by gender, the 3 studies conducted with all male participants (29,31,32) had a larger effect size (−3.82 kg) and minimal heterogeneity (*I* ^2^ = 0%) compared with the 8 studies with more than 75% female participants (24–28,34–36) (−2.06 kg; *I* ^2^ = 69%) and the 3 studies with a more balanced mix of male and female participants (−3.16 kg; *I* ^2^ = 0%) (30,33,37). Baseline average age and baseline weight were similar across studies, so we do not report results by these subgroups. Intervention focus was also similar across studies (ie, most interventions focused on nutrition and exercise), so we do not report results by these characteristics.

We also conducted a sensitivity analysis that included alternative intervention arms for studies with more than 1 intervention group (25,27–30,34,36,37). The mean difference in pooled weight loss was slightly smaller than that of the selected primary intervention arm overall (−2.10 kg, 95% CI, −2.92 to −1.28) and by intervention time point (−2.16 kg; 95% CI, −3.02 to −1.31 for 13 weeks duration and −2.05 kg; 95% CI, −4.11 to 0 for 13–26 weeks duration). Heterogeneity was substantial overall (*I* ^2^ = 69%) and for interventions of less than 13 weeks (*I* ^2^ = 67%) and 13 to 26 weeks (*I* ^2^ = 73%).

## Discussion

This meta-analysis of 14 RCTs found that interventions lasting 6 months or less were effective at achieving weight loss. Each study showed weight loss relative to control groups. The pooled mean difference in weight change was −2.59 kg compared with controls but may be further diminished when interventions are translated into real-world practice. However, adults with overweight and obesity tend to gain weight over time in the real world (eg, ~1% for >6 y) (38), such that lifestyle change interventions that slow or reverse weight gain trajectories are important in reducing the risk for developing chronic diseases. Thus, a key contribution of our study is bolstering the evidence that short-term lifestyle change interventions may result in weight change benefits in adults with overweight or obesity and could provide an alternative to longer interventions that some people may be unable or unwilling to complete (8,39,40). However, we do not know whether participants in these short-term interventions benefit, either in terms of weight change or chronic disease prevention. Our findings may have important health implications. Although the mean difference of approximately 2 kg among participants in the lifestyle change interventions relative to controls is modest, it can be clinically meaningful, because a lifestyle modification RCT reported a 16% reduction in 3-year diabetes risk for every kilogram of weight loss in the intervention group through lifestyle change (41).

All lifestyle interventions included in our meta-analysis were multicomponent, which may aid weight loss. This is consistent with findings from a recent meta-analysis where authors found that overall multicomponent lifestyle interventions were effective at achieving weight loss ranging from −1.3 kg to −8.2 kg at 5 to 6 months (42). The interventions included in that meta-analysis used various components to promote weight loss. The most frequent intervention components were educational resources, followed by support tools, such as pedometers and food and exercise diaries. Such components may facilitate self-monitoring of diet and body weight, which other studies have shown is a key to achieving healthy lifestyle behaviors (43) and preventing regain of weight lost (44). Our analysis did not examine which intervention components individually contributed to weight change. However, a recent systematic review and meta-analysis assessed the contribution of individual intervention components of lifestyle change programs, finding that change in diet, offering partial or total meal replacements, delivery by a psychologist–counselor or dietitian, and delivery in a home setting were associated with significant benefit in weight change (45). Additional research may be needed to disentangle the intervention components that drive weight change for interventions of shorter durations, such as the type of dietary guidance or the frequency and nature of physical activity recommendations. Additionally, future work should explore how social determinants of health, such as access to affordable and quality healthy food or safe places for physical activity, affect program and health outcomes (46). Understanding how different components of weight loss interventions can be adapted, tailored, or enhanced in response to contextual social determinants of health factors will help to ensure these types of interventions are equitable and accessible. Finally, 12 of the 14 included studies focused on improving both nutrition and physical activity to achieve weight loss. Therefore, we were unable to compare the effect of weight-loss interventions focused on nutrition alone versus physical activity alone. However, 1 systematic review and meta-analysis suggested that lifestyle change interventions that involved both diet and physical activity were associated with greater weight loss than those focused on diet (mean difference: –1.72 kg) or physical activity (mean difference: −5.33 kg) alone (47).

An important finding of our meta-analysis is that the interventions that lasted less than 13 weeks appear to be at least as effective for weight loss as those lasting from 13 to 26 weeks. One possible explanation for this finding is that interventions with a shorter duration showed a greater retention rate (~80%) than interventions of longer duration (~70%) in our analysis. This is consistent with other work that reported that programs of longer duration may experience higher dropout rates (48). In turn, high retention was important to increase weight loss from ~2 kg to ~4 kg in our sensitivity analysis where high attrition studies were removed, even when the intervention was relatively short in duration. This finding emphasizes that among interventions similar in length where higher retention is correlated with more significant weight loss (49,50), the success of these interventions also depends on sustained participant engagement. Future research should focus on determining which elements, such as personalized feedback or flexible scheduling, enhance retention.

Findings related to the effect of intervention duration in other meta-analyses are mixed. For example, 1 prior meta-analysis demonstrated that interventions lasting 12 months or more yielded slightly more weight loss for people with overweight or obesity compared with interventions lasting 6 months or less (15), whereas other meta-analyses reported no difference in weight loss by intervention duration (51,52). Nonetheless, interventions that require long-term engagement from participants may preclude some people from ever enrolling (53). Lengthier interventions can also be more challenging to disseminate and sustain because of the burden they place on the organizations that provide them (48).

### Limitations

Our review has some limitations. First, in our meta-analysis we examined only weight change at the end of the intervention period and did not analyze any follow-up weight change that may have been reported; therefore, we could not make any conclusions about the ability of short-term interventions to sustain weight loss or reduce diabetes risk. Additional research could examine the effect of short-term interventions on sustained weight loss. Although 7 studies had substantial dropout rates at the end of the intervention (24–26,33,35–37), our sensitivity analysis showed that excluding these studies did not substantively change the overall findings. Also, given the multicomponent nature of nearly all the interventions we examined, we were unable to conclude which specific components are essential to driving weight loss. Future work should aim to disentangle the intervention components that may drive weight change for interventions of short duration. Although we improved heterogeneity by grouping studies according to their comparison group, weight loss possibly may be affected by other factors that vary between studies, such as different intensity and frequency of the interventions or differences in participant characteristics. For example, the included studies contained limited or no information on participant race or ethnicity and socioeconomic status, and some populations might respond differently to lifestyle interventions. Ensuring that interventions reduce existing health inequities is important but can be a challenge with long-term interventions that have resulted in better weight loss outcomes for participants who are non-Hispanic White and of higher socioeconomic status (39).

### Conclusion

Short-term multicomponent interventions can possibly be effective in achieving clinically significant weight loss for adults with overweight or obesity. Participating in longer interventions may lead to more substantial results but may not be feasible for some people because of work schedules, caregiving responsibilities, transportation requirements, or other factors. Our findings can be used to inform a person’s decision making when offered a choice of programs, and by clinicians and researchers who can continue developing short-term alternatives to long interventions. Providing both short- and long-term options could increase opportunities for people to begin lifestyle changes and facilitate their choosing a program that best suits their schedule, needs, and available resources.
